# Myelin-associated proteins are potential diagnostic markers in patients with primary brain tumour 

**DOI:** 10.1080/07853890.2021.1983205

**Published:** 2021-10-03

**Authors:** Olga M. Koper-Lenkiewicz, Anna J. Milewska, Joanna Kamińska, Karol Sawicki, Robert Chrzanowski, Justyna Zińczuk, Joanna Reszeć, Marzena Tylicka, Ewa Matuszczak, Joanna Matowicka-Karna, Zenon Mariak, Mariusz W. Mucha, Robert Pawlak, Violetta Dymicka-Piekarska

**Affiliations:** a Department of Clinical Laboratory Diagnostics, Medical University of Bialystok, Białystok, Poland; b Department of Statistics and Medical Informatics, Medical University of Bialystok, Białystok, Poland; c Department of Neurosurgery, Medical University of Bialystok, Białystok, Poland; d Department of Medical Pathomorphology, Medical University of Bialystok, Białystok, Poland; e Department of Biophysics, Medical University of Białystok, Białystok, Poland; f Department of Pediatric Surgery, Medical University of Białystok, Białystok, Poland; g Institute of Biomedical and Clinical Science, Hatherly Laboratories, University of Exeter Medical School, Exeter, UK

**Keywords:** Biomarker, cerebrospinal fluid, myelin-associated proteins, primary brain tumour

## Abstract

**Introduction:**

Taking into account the possibility of myelin-associated proteins having a role in brain tumour development, the study aimed to evaluate the diagnostic usefulness of myelin-associated proteins (Nogo-A, MAG, OMgp) released into extracellular space in patients with brain tumours.

**Patients and methods:**

Protein concentration in primary brain tumour (*n* = 49) and non-tumoural subjects (*n* = 24) was measured in cerebrospinal fluid (CSF) and serum by means of ELISA. Immunohistochemistry for IDH1-R132H was done on 5-μm thick formalin-fixed, paraffin-embedded tumour sections with the use of an antibody specific for the mutant IDH1-R132H protein.

**Results:**

The receiver operator characteristic curve analysis showed that CSF Nogo-A and serum MAG were useful in differentiating patients with primary brain tumour from non-tumoural individuals. This was also true in the case of the separate analysis of the astrocytic tumour versus non-tumoural groups and the meningeal tumour versus non-tumoural groups. Neither Nogo-A nor MAG or OMgp concentrations were significantly different, in serum or CSF, between *IDH1* wild-type astrocytic brain tumour patients compared to *IDH1* mutant patients.

**Conclusions:**

Our results indicated the potential usefulness of CSF Nogo-A and serum MAG evaluation as circulating biomarkers of primary brain tumours. Because blood is relatively easy to obtain, future research should be conducted to explicitly indicate the value of serum MAG concentration evaluation as a brain tumour biomarker.

**Key messages**
1. Myelin-associated proteins may be circulating brain tumour biomarkers.
2. Nogo-A and MAG proteins seem to be the most useful in brain tumour diagnosis.
3. Decreased CSF Nogo-A concentration is an adverse prognostic factor for patients’ survival.

## 1. Introduction

Despite the dramatic development in neuroimaging techniques in recent years, the issue of early diagnosis of malignant glial brain tumours persists. Glioma is usually detected too late, when it has infiltrated the brain to a major extent, thus preventing early intervention. The discovery of a circulating biomarker for these tumours would be of great importance [1–4].

Neurite growth isoform A of reticulon-4 (Nogo-A), myelin-associated glycoprotein (MAG), and oligodendrocyte myelin glycoprotein (OMgp) are all myelin-derived proteins. Nogo-A expression is found in neurons and oligodendrocytes of the central nervous system in adults [5]. MAG is present in the innermost membrane of the mature, compact myelin of astrocytes, oligodendrocytes, and Schwann cells. This minor myelin component is present in both the central and peripheral nervous system [6]. Interestingly, MAG has a dual function; in embryonic neurons it promotes axonal growth, whereas in adult neurons it inhibits axonal growth [7]. OMgp is a glycosylphosphatidylinositol (GPI)-anchored protein expressed in the central nervous system in both neurons and oligodendrocytes. At the NODES of Ranvier, OMgp mediates the oligodendrocyte–oligodendrocyte and oligodendrocyte–axonal membrane interactions [6].

After the injury, such as those occurring during glioma invasion, Nogo-A, MAG, and OMgp are released from the cell membrane to the extracellular space. Their growth-inhibitory signal is transmitted via the cell surface receptor complex including Nogo Receptor 1 (NgR1), Lingo-1, p75NTR, and TROY. The recently paired immunoglobulin receptor B protein (PirB) has also been identified as an additional transmembrane receptor with a high affinity to bind myelin-associated proteins [6].

Although the involvement of Nogo-A, MAG, and OMgp in the inhibition of central nervous system regeneration has been widely studied [6], the data on their role in brain tumour pathophysiology is scarce. The few existing studies concern mostly Nogo-A [8–12] and only one study focuses on MAG [13]. Our previous work was the only one to evaluate Nogo-A concentration in patients with primary brain tumours – its cerebrospinal fluid (CSF) levels were significantly lower compared to non-tumoural subjects [12]. The extracellular pool of Nogo-A and MAG could be of principal importance for glioma growth as NgR1 activation by these proteins inhibits glioma cell motility and invasiveness *in vitro* [13]. The role of OMgp in brain tumours has not been studied.

Taking into account the possibility of myelin-associated proteins having a role in brain tumour development, the study aimed to evaluate whether CSF and serum MAG and OMgp concentrations are different between astrocytic and meningeal tumour patients compared to non-tumoural individuals. In the next step, we compared the diagnostic utility of evaluating the three myelin-associated proteins of interest (Nogo-A, MAG, OMgp) in patients with primary brain tumour. Because isocitrate dehydrogenase (*IDH*) glioma mutants showed a better prognosis compared to *IDH*-wildtype glioma patients, in the astrocytic brain tumour subgroup we also analyzed Nogo-A, MAG, and OMgp levels depending on the *IDH1* gene mutation. Finally, we examined the relationship between the tested proteins and patient diagnosis and survival.

## 2. Patients and methods

### 2.1. Subjects

The study population included 49 individuals (25 males/24 females; median age 59 years, with an age range of 36–83 years) with previously untreated primary brain tumours: patients with astrocytic brain tumours (*N* = 33) and patients with tumours of the meninges (*N* = 16). For individual patient characteristics see Supplementary Materials (Table S1). A brain tumour remission in medical history was the exclusion criterion. The comparative group was composed of 24 non-tumoural subjects (5 males/19 females; median age 59 years, with an age range from 30 to 70 years) with an unruptured intracranial aneurysm, which is usually asymptomatic [14] and discovered incidentally [14,15]. The exclusion criteria included: cancer in medical history or acute/chronic inflammatory diseases. Patients with brain tumour and control subjects were recruited between July 2015 and March 2017.

**Table 1.** Nogo-A, MAG, and OMgp concentrations in patients with brain tumours compared to non-tumoural individuals.

	Total brain tumour	Astrocytic brain tumour	Meningeal tumour	Non-tumoural group
	Nogo-A (pg/mL)			
CSF	418.00 (249.00–1964.00)^b^	462.00 (352.00–2998.00)^a,b^	234.00 (98.50–646.50)^b^	6,550.00 (3,627.00–10,017.00)
Serum	0.00 (0.00–0.00)	0.00 (0.00–0.00)	0.00 (0.00–0.00)	0.00 (0.00–0.00)
	MAG (ng/mL)			
CSF	7.43 (4.15–12.99)	7.12 (2.77–16.58)	7.43 (6.22–9.15)	9.86 (6.67–13.92)
Serum	0.00 (0.00–2.65)^b^	0.00 (0.00–4.18)^b^	0.00 (0.00–2.61)^b^	6.95 (3.03–14.19)
	OMgp (ng/mL)			
CSF	0.00 (0.00–0.09)	0.00 (0.00–0.80)	0.00 (0.00–0.00)	0.00 (0.00–0.00)
		AR = 23.1	AR = 18.1	AR = 16.0
Serum	0.00 (0.00–0.10)^b^	0.00 (0.00–0.46)	0.00 (0.00–0.00)^b^	0.24 (0.00–1.49)
		AR = 20.3	AR = 14.1	AR = 27.4

Results are presented as median with 25th and 75th percentiles. For patients with astrocytic brain tumour, patients with meningeal brain tumour, and control subjects we also calculated the OMgp average rank (AR), which better characterizes concentration changes between these groups.

CSF: cerebrospinal fluid.

a Statistically significant when compared to the meningeal group.

^b^Statistically significant vs. non-tumoural group.

### 2.2. Sample collection and storage

At the Department of Neurosurgery at the Clinical Medical Hospital in Bialystok, CSF and serum samples were procured. CSF was extracted from the subarachnoid space of the brain, during a craniotomical procedure. Surgical procedures were performed conventionally: under general anaesthesia with a three-pin Mayfield headholder to fix the patient’s head in position. Lifting of the bone flap was preceded by skin incision and lancing of dura mater, thus permitting visualization of the arachnoid membrane and subarachnoid space. With the assistance of an operating microscope, the subarachnoid space was opened carefully and inflowing CSF was aspirated with a single-use, sterile syringe, and soft venous catheter. Particular attention was paid to preventing contamination of the CSF with blood or the warm saline solution used as irrigation. Thus, each of the aforementioned steps was carried out at the very beginning of each procedure, before any bleeding might have occurred [16].

Patients’ blood was collected in 2.7-mL test tubes without anticoagulant (S-Monovette, SARSTEDT). CSF and blood samples were centrifuged (20 min/1000*g*) within 0.5 h after collection. Obtained serum and CSF supernatant samples were aliquoted and stored at –75 °C until further analysis.

The study was conducted in agreement with the Helsinki-II-declaration and was approved by the Bioethics Human Research Committee of the Medical University of Bialystok (Permission No. R-I-002/383/2015). Informed consent was obtained from all subjects involved in the study.

### 2.3. Nogo-A, MAG, and OMgp concentration analysis

To determine whether myelin-associated protein concentration changed in individuals with brain tumours, we first evaluated protein levels in CSF and serum. Nogo-A concentration was analyzed in 49 patients with brain tumour and 24 non-tumoural individuals. MAG and OMgp concentrations were analyzed in 30 patients with brain tumour and 10 non-tumoural subjects.

Nogo-A, MAG, and OMgp concentrations were analyzed by means of the enzyme-linked immunosorbent assay (ELISA) method in compliance with the manufacturer’s instructions. CSF and serum samples were not diluted before analysis.

Nogo-A concentration was measured using a Human RTN4 ELISA kit (Catalogue No. EH3732) from Wuhan Fine Biological Technology Co., Ltd. The detection range for the kit is between 78.125 and 5000 pg/mL, the minimum detectable dose (sensitivity) of the assay is <46.875 pg/mL. The sensitivity of this assay was defined as the lowest protein concentration that could be differentiated from 0.00 pg/mL. It was determined by the mean O.D. value of 20 replicates of the zero standard added to their three standard deviations (SD). Values <46.875 ng/mL were considered as 0.00 ng/mL.

MAG concentration was measured using the Human MAG ELISA kit (Catalogue No. orb407125) from Biorbyt Ltd., Cambridge, England. The detection range for the kit is between 0.625 and 40 ng/mL, the sensitivity of the assay is 0.156 ng/mL. The sensitivity of this assay was defined as the lowest protein concentration that could be differentiated from 0.00 pg/mL. It was determined by the mean O.D. value of 20 replicates of the zero standard added to their three standard deviations (SD). Values <0.156 ng/mL were considered as 0.00 ng/mL.

OMgp concentration was measured using the Human OMG ELISA kit (Catalogue No. orb404974) from Biorbyt Ltd., Cambridge, England. The detection range for the kit is between 0.156 and 10 ng/mL, the sensitivity of the assay is 0.039 ng/mL. The sensitivity of this assay was defined as the lowest protein concentration that could be differentiated from 0.00 pg/mL. It was determined by the mean O.D. value of 20 replicates of the zero standard added to their three standard deviations (SD). Values <0.039 ng/mL were considered as 0.00 ng/mL.

### 2.4. *IDH1* mutation detection

*IDH1* mutation, representing above 90% of the IDH mutations existing in gliomas [14], is detected by R132H monoclonal specific antibodies. Immunohistochemistry for IDH1-R132H was done on 5-μm thick formalin-fixed, paraffin-embedded tumour sections. A specific antibody for the mutant IDH1-R132H protein (H09, Dianova, dil 1:100) was applied. As a detection system, a labelled streptavidin-biotin kit (Agilent, Denmark) was used. Antigen retrieval was performed in citrate buffer (pH 6.0) in a pTLink (Agilent).

Combined cytoplasmic and nuclear staining was interpreted as immunopositive. Three-tiered semiquantitative system results were as follows: if no tumour cell was immunopositive – it was reported as negative; if there was an admixture of immunopositive and immunonegative tumour cells or areas of immunopositive and immunonegative tumour cells were adjacent to each other – it was reported as partly/focal positivity; if all tumour cells were immunopositive – it was reported as complete (diffuse) positivity.

### 2.5. Statistical analysis

The obtained results were statistically analyzed with the use of the STATISTICA 12.0 PL software (StatSoft Inc., Tulsa, USA), STATA 12.1 (StataCorp LP), and GraphPad Prism 5.0 (GraphPad Software, San Diego, USA). The concentration of the tested parameters did not follow a normal distribution (Shapiro–Wilk test), thus nonparametric statistical analysis was employed. The Mann–Whitney test was used to compare two independent samples, and the ANOVA rank Kruskal–Wallis test was used for the comparison of three samples. Values for measured variables are stated as median with 25th and 75th percentiles (interquartiles, IQs). Receiver operator characteristic (ROC) curve was generated to calculate the area under the ROC curve (AUC) [17,18]. The Youden index, which is a function of sensitivity and specificity, was estimated to indicate an optimal cut-off value [19]. Logistic regression analysis was performed to find out whether the concentration of tested molecules may be predictive in brain tumour diagnosis. In the whole study group, we also performed a Kaplan–Meier survival analysis. Differences were considered statistically significant for two-tailed *p* < .05.

## 3. Results

### 3.1. CSF Nogo-A, MAG, and OMgp results

**Figure 1.**
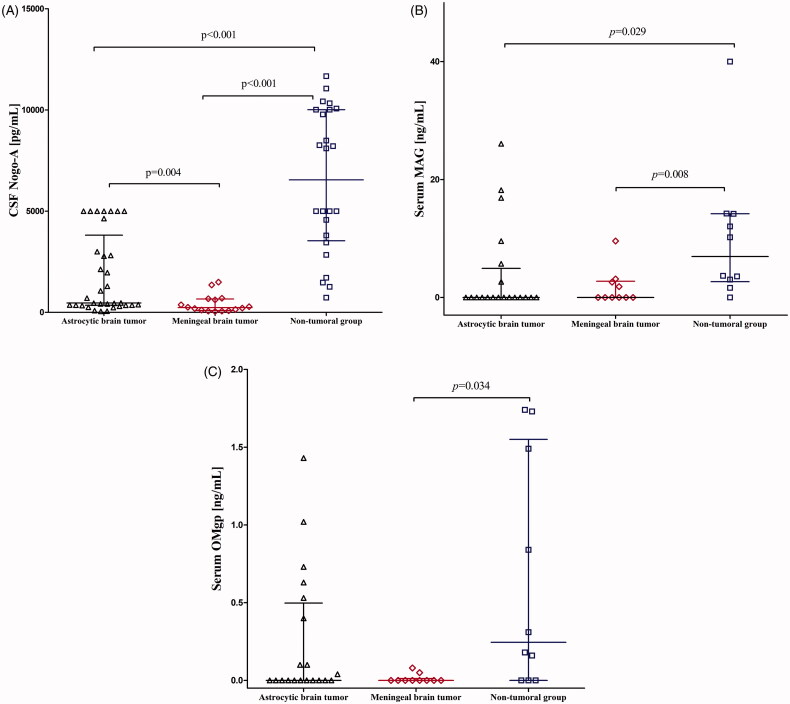
(A) CSF Nogo-A dot plots for the astrocytic and meningeal brain tumour groups and non-tumoural individuals. (B) Serum MAG dot plots for the astrocytic and meningeal brain tumour groups and non-tumoural individuals. (C) Serum OMgp dot plots for the astrocytic and meningeal brain tumour groups and non-tumoural individuals. *p* < .05 is considered to be statistically relevant. CSF: cerebrospinal fluid.

Overall, the group of patients with brain tumour had statistically lower CSF Nogo-A concentrations when compared to non-tumoural subjects (*p* < .001). ANOVA rank Kruskal–Wallis indicated that both, astrocytic and meningeal tumour subgroups had statistically lower CSF Nogo-A concentrations compared to non-tumoural individuals (*H* = 37.57; df = 2; *p* < .001, respectively) (Table 1 and Figure 1(A)).

CSF MAG results were below the sensitivity of the assay kit in one patient with meningeal brain tumour. Overall, the group of patients with brain tumour had lower CSF MAG concentrations compared to the non-tumoural group, but it was not significant (*p* = .508). The ANOVA rank Kruskal–Wallis test did not show a statistical difference for CSF MAG concentrations (*H* = 0.49; df = 2; *p* = .780) (Table 1).

CSF OMgp results were below the sensitivity of the assay kit in thirteen patients with astrocytic brain tumour, eight patients with meningeal brain tumour, and all non-tumoural individuals. Overall, the group patients with of brain tumour had the same CSF OMgp median concentration as non-tumoural subjects. Also, the ANOVA rank Kruskal–Wallis test did not show a statistical difference for CSF OMgp concentrations (*H* = 5.86; df = 2; *p* = .053) (Table 1).

Altogether the aforementioned data demonstrate that from all the tested proteins only CSF Nogo-A significantly differentiated patients with brain tumour from non-tumoural individuals. Thus for further diagnostic utility, analysis of only CSF Nogo-A was pursued.

### 3.2. Serum Nogo-A, MAG, and OMgp results

In both patients with brain tumour and non-tumoural individuals, serum Nogo-A concentrations were undetectable. Serum MAG results were below the sensitivity of the assay kit in fourteen patients with astrocytic brain tumour, six patients with meningeal brain tumour, and one non-tumoural individual. The combined brain tumour groups had statistically lower serum MAG concentrations compared to the non-tumoural group (*p* = .004). ANOVA rank Kruskal–Wallis showed a statistically significant difference between patients with astrocytic brain tumour and non-tumoural individuals (*H* = 9.05; df = 2; *p* = .029) as well as between patients with meningeal tumour and non-tumoural individuals (*p* = .008) (Table 1 and Figure 1(B)).

Serum OMgp results were below the sensitivity of the assay kit in eleven patients with astrocytic brain tumour, nine patients with meningeal brain tumour, and three non-tumoural individuals. The combined brain tumour groups had statistically lower serum OMgp concentrations compared to the non-tumoural group (*p* = .031). The ANOVA rank Kruskal–Wallis test showed a statistically significant difference between patients with meningeal brain tumour and non-tumoural individuals (*H* = 7.72; df = 2; *p* = .034) (Table 1 and Figure 1(C)).

The aformentioned data demonstrate that MAG and OMgp could both be potentially useful biomarkers in the serum. However, for further diagnostic utility analysis, we decided to pursue only serum MAG. The reason for our decision is presented in the Discussion section.

### 3.3. CSF MAG versus serum MAG

To investigate whether MAG expression differs between the central and peripheral nervous system, we compared CSF to serum values. Patients with brain tumour had significantly higher median CSF MAG concentrations compared to serum MAG values (7.43 ng/mL vs. 0.00 ng/mL; *p* < .001). On the contrary, in the non-tumoural group median CSF MAG concentration was not statistically different compared to the serum value (9.86 ng/mL vs. 6.95 ng/mL; *p* = .436). These results indicate differing serum MAG expressions in patients with brain tumour and non-tumoural subjects.

### 3.4. Nogo-A, MAG, and OMgp concentrations depending on the IDH1 mutation

Because the available literature indicates that isocitrate dehydrogenase (*IDH*) glioma mutants showed a better prognosis compared to *IDH*-wildtype glioma patients at different WHO grades [20,21], we also analyzed Nogo-A, MAG, and OMgp levels depending on the *IDH1* gene mutation in the astrocytic brain tumour subgroup. Neither Nogo-A nor MAG or OMgp concentrations were significantly different, in serum or CSF, between patients with *IDH1* wild-type astrocytic brain tumour compared to patients with *IDH1* mutant astrocytic brain tumour (data not shown), which may indirectly indicate that the tested molecules could not be recognized as prognostic biomarkers of brain tumours.

### 3.5. Logistic regression analysis results

To find out whether the concentrations of Nogo-A, MAG, and OMgp may predict brain tumour diagnosis, we conducted logistic regression analysis. In the model of univariate logistic regression analysis, predictor variables influencing brain tumour diagnosis included: sex, CSF Nogo-A concentration, white blood cell count (WBC), potassium (K+), glucose, and urea concentrations. We showed that: (1) the chances of developing a brain tumour in men are almost four times higher than in women; (2) with an increase in CSF Nogo-A by 100 pg/mL, the chances of a brain tumour decrease by 6.11%; (3) with an increase in WBC by 1 × 10^3^/µL, the chances of brain tumour presence increase by almost one and a half times; (4) with an increase in K + concentration by 1 mg/dL, the chances of brain tumour presence increase by 3.71-fold; (5) with an increase in glucose concentration of 10 mg/dL, the chances of brain tumour presence increase by 25.37%; and (6) with an increase in urea concentration by 1 mg/dL, the chances of brain tumour presence increase by 9.00% (Table 2).

**Table 2.** Logistic regression analysis.

Covariates	OR	95% CI	*p* Value
Univariate logistic regression analysis			
Sex	3.958	1.27–12.29	.017
CSF Nogo-A (pg/mL)	0.999^a^	0.99–0.99	<.001
WBC (×10^3^/µL)	1.446	1.17–1.78	.001
K^+^ (mmol/L)	3.709	1.09–12.57	.035
Glucose (mg/dL)	1.022^b^	1.00–1.04	.034
Urea (mg/dL)	1.090	1.04–1.14	.001
Multivariate logistic regression analysis			
CSF Nogo-A (pg/mL)	0.998^a^	0.99–0.99	.005
K^+^ (mmol/L)	17.264	1.21–246.73	.036
Urea (mg/dL)	1.228	1.05–1.44	.010
Creatinine (mg/dL)	0.001	0.00–0.70	.039

OR, odds ratio; CI, confidence interval.

a OR interpreted by intervals of 100 units.

b OR interpreted by intervals of 10 units.

Conversion factor conventional to SI unit: WBC [10^9^/L] – 1.0, glucose [mmol/L] – 0.0555, urea [mmol/L] – 0.357, creatinine [µmol/L] – 88.402.

In the model of multivariate logistic regression analysis, predictor variables influencing brain tumour diagnosis included: CSF Nogo-A, K+, urea, and creatinine concentrations. We showed that: (1) with an increase in CSF Nogo-A by 100 pg/mL, the chances of brain tumour presence decrease by 15.03%; (2) with an increase in K + concentration by 1 mg/dL, the chances of brain tumour presence increase by more than 17-fold; (3) with an increase in urea concentrations by 1 mg/dL, the chances of brain tumour presence increase by 23.00%; (4) with an increase in creatinine concentration by 0.01 mg/dL, the chances of developing a brain tumour decrease by 6.76% (Table 2).

### 3.6. CSF Nogo-A and serum MAG diagnostic utility analysis

**Figure 2.**
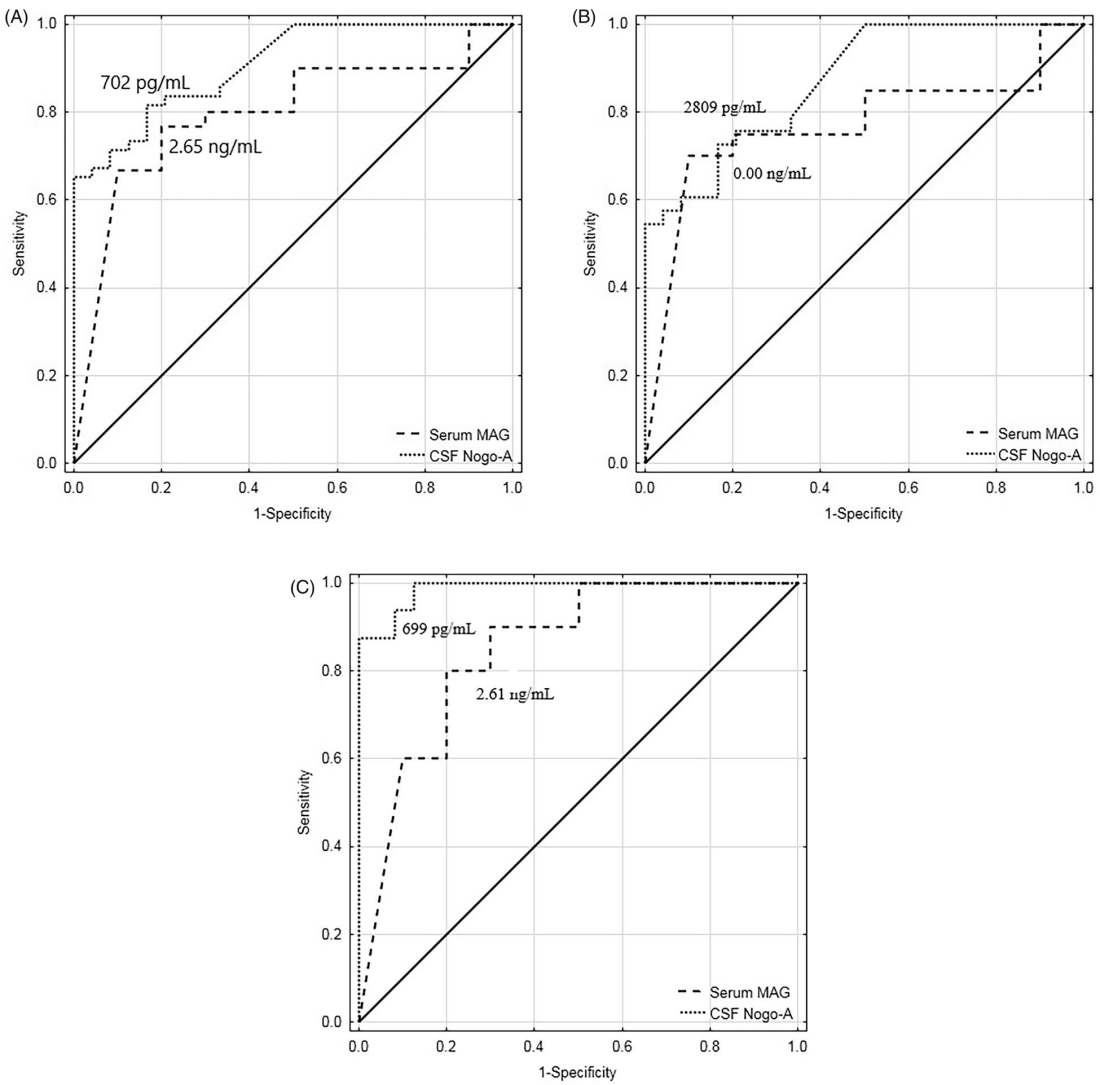
(A) ROC curves for CSF Nogo-A (AUC ± SE = 0.909 ± 0.033, *p* < .001; YI = 0.65; SE = 65%; SP = 100%; AC = 77%; PPV = 100%; NPV = 59%) and serum MAG (AUC ± SE = 0.797 ± 0.079, *p* < .001; YI = 0.57; SE = 77%; SP = 80%; AC = 78%; PPV = 92%; NPV = 53%) for differentiating the combined brain tumour groups from non-tumoural individuals. (B) ROC curves for CSF Nogo-A (AUC ± SE = 0.871 ± 0.045, *p* < .001; YI = 0.56; SE = 73%; SP = 83%; AC = 77%; PPV = 86%; NPV = 69%) and serum MAG (AUC ± SE = 0.770 ± 0.090, *p* = .003; YI = 0.60; SE = 70%; SP = 90%; AC = 77%; PPV = 93%; NPV = 60%) for differentiating the astrocytic brain tumour group from non-tumoural individuals. C: ROC curves for CSF Nogo-A (AUC ± SE = 0.987 ± 0.013, *p* < .001; YI = 0.88; SE = 88%; SP = 100%; AC = 95%; PPV = 100%; NPV = 92%) and serum MAG (AUC ± SE = 0.850 ± 0.089, *p* < .001; YI = 0.60; SE = 80%; SP = 80%; AC = 80%; PPV = 80%; NPV = 80%) for differentiating the meningeal brain tumour group from non-tumoural individuals. ROC: receiver operating characteristic; CSF: cerebrospinal fluid; AUC: area under the ROC curve; SE: standard error; Cut-off: optimal cut-off based on the highest Youden Index; YI: Youden Index; SE: sensitivity; SP: specificity; AC: diagnostic accuracy; PPV: positive predictive value; NPV: negative predictive value.

The diagnostic utility analysis found that both CSF Nogo-A and serum MAG were useful in differentiating patients with primary brain tumour from non-tumoural individuals. This was also true in differentiating individual brain tumour subgroups, patients with astrocytic brain tumour, or patients with meningeal brain tumour, from non-tumoural subjects. Our findings present CSF Nogo-A and serum MAG as being potentially useful circulating biomarkers of primary brain tumours (Figure 2(A–C)).

### 3.7. Kaplan–Meier survival analysis for the whole study group

**Figure 3.**
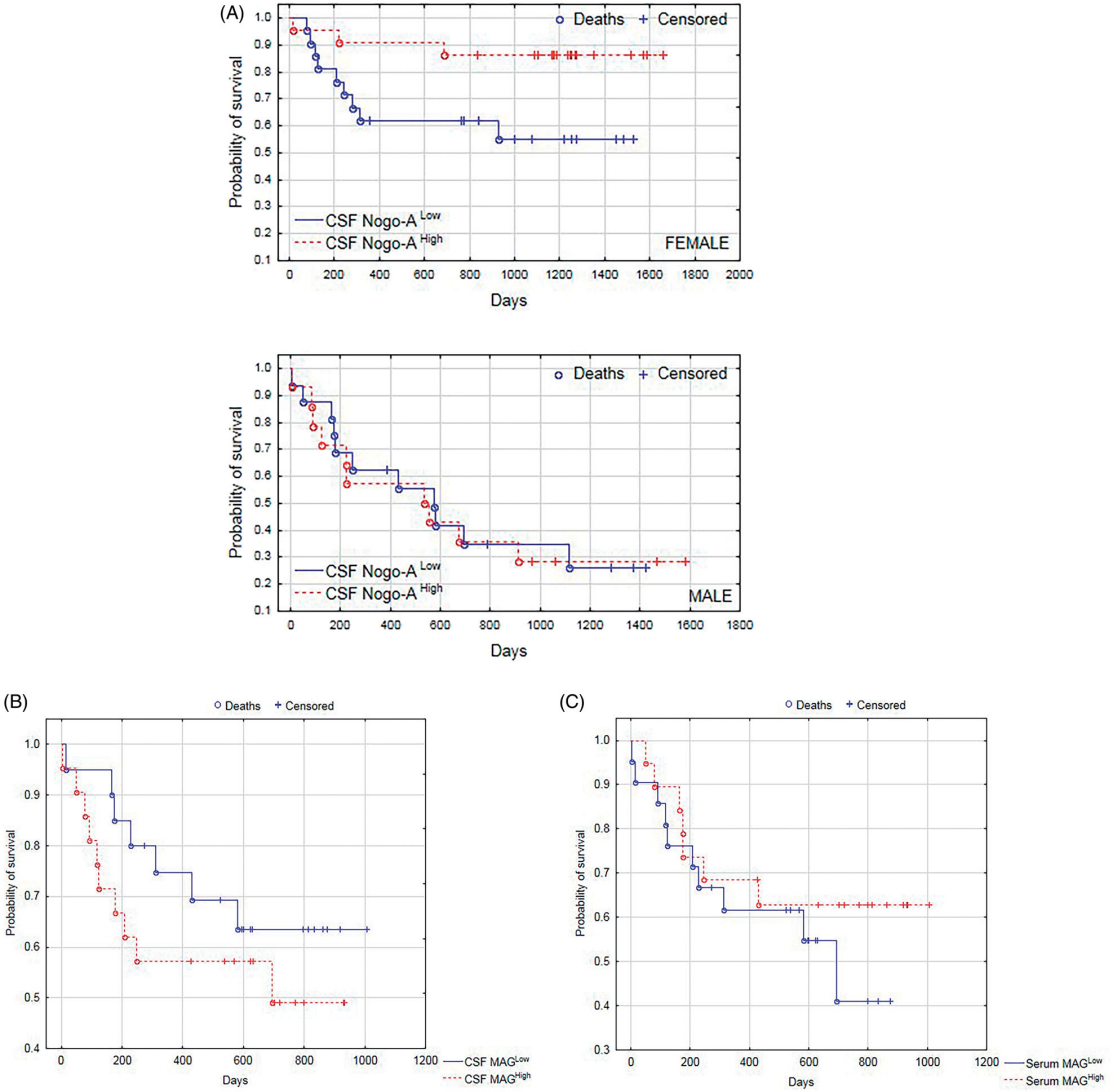
**(**A) Kaplan–Meier survival analysis for the total study group. Males and females were analyzed separately. Based on CSF Nogo-A median concentrations, males/females were divided into those with CSF Nogo-A^Low^ (≤median; Me = 1356 pg/mL) and those with CSF Nogo-A^High^ (>median). (B) Kaplan–Meier survival analysis for the whole study group. Based on CSF MAG median concentrations, patients were divided into those with CSF MAG^Low^ (≤median; Me = 7.43 ng/mL) and those with CSF MAG^High^ (>median). (C) Kaplan–Meier survival analysis for the whole study group. Based on serum MAG median concentrations, patients were divided into those with serum MAG^Low^ (≤median; Me = 0.00 ng/mL) and those with serum MAG^High^ (>median). CSF, cerebrospinal fluid.

Kaplan–Meier survival analysis was performed for the whole study group. The survival status was updated in February 2020. We found that the probability of survival showed a tendency to be greater for patients with higher CSF Nogo-A concentrations, but it was not significant (*p* = .102). Interestingly, when separately analyzing males and females, we found that the probability of survival was significantly greater for females with higher CSF Nogo-A concentrations (*p* = .032, Figure 3(A)). Intriguingly, the probability of survival showed a tendency to be greater for patients with a lower CSF MAG concentration; it was visible but not significant (*p* = .318, Figure 3(B)). Kaplan–Meyer curve analysis showed that the probability of survival tended to be greater for patients with higher serum MAG concentrations compared to those with lower serum MAG concentrations, but the obtained difference was not significant (*p* = .429, Figure 3(C)).

## 4. Discussion

Our study is the first which evaluated MAG and OMgp concentrations in CSF and serum of patients with primary brain tumours. We showed that both serum MAG and serum OMgp concentrations in the combined brain tumour groups were statistically lower compared to the non-tumoural group. This was also true when analyzing the brain tumour subgroups separately (astrocytic and meningeal brain tumour groups).

An unexpected result was the finding of some serum OMgp concentrations in both primary brain tumours (*N* = 11) and non-tumoural individuals (*N* = 7). This is interesting because according to the literature OMgp is exclusively expressed only within the central nervous system [6]. The primary structure of OMgp is composed of four domains. At the N-terminus there is 32 aa Cys-rich motif (CR), which is followed by a 7 ½ tandem Leucine-rich repeats (LRRs) of 24 aa each, and subsequently by a domain of 4 ½ repeats of 40 residues each rich in Ser/Thr. The COOH-terminus contains glycosylphosphatidylinositol (GPI) as a membrane anchor. Among proteins, the platelet glycoprotein Ib (GPIb) was found to be the most similar to OMgp. These two proteins share richly in CR and LRRs motifs, but also contain Ser/Thr-rich regions [22]. Moreover, Mikol et al. [22] showed the similarity between the GPIbαβ heterodimer gene and the OMgp gene, which indicates that these two proteins may also be related by gene structure. The possible cross-reactivity in the ELISA test between these two proteins could account for our current results. For this reason, the obtained results regarding OMgp concentrations will not be subjected to further discussion.

Our current study presented CSF Nogo-A concentration as statistically lower in the brain tumour group compared to non-tumoural subjects. This was also true for both astrocytic brain tumours and meningeal tumours. Serum Nogo-A concentrations using the ELISA method were not detected in both brain tumours and non-tumoural subjects. This is in line with already published results indicating that Nogo-A is exclusively expressed within the central nervous system [6].

Studies of Jung et al. [10] and Kuhlmann et al. [11] showed significantly higher Nogo-A expression in oligodendroglial tumours compared to astrocytic tumours, which could be explained by the fact that Nogo-A protein is found only in neurons and oligodendrocytes [5]. It is absent in the meninges of the mature adult brain [23]. Our study group of patients with neuroepithelial brain tumour was composed exclusively of astrocytic brain tumours. Altogether, this data could explain the decreased CSF Nogo-A concentrations in the current cohort of patients with brain tumour (astrocytic plus meningeal) compared to non-tumoural individuals, and moreover strengthen the hypothesis that decreased CSF Nogo-A could be considered a marker for these tumours.

We found that serum MAG concentration in patients with primary brain tumour was significantly lower compared to non-tumoural individuals. MAG is present in the myelin of Schwann cells [6]. Thus, we suggest that MAG expression within myelin of the peripheral nervous system in patients with primary brain tumour may be lower compared to non-tumoural individuals.

Liao et al. [13] showed that the adhesiveness of U87MG cells is inhibited via the interaction of MAG and Nogo-66 with the NgR on the plasma membrane of these cells. In further studies, Jin et al. [8] transfected U87MG cells with a sense-Nogo-A cDNA construct and compared them to U87MG cells not expressing Nogo-A (U87MG-E cells). They found that the migration, invasiveness, Rho activity as well as phosphorylated cofilin expression of the U87MG-Nogo-A cells was decreased compared to the control ones. Moreover, U87MG-Nogo-A cells became flatter than those from the U87MG-E line. The authors suggested that Nogo-A may inhibit migration and invasiveness of glioma cells by decreased RhoA-cofilin signalling [8]. Taking into consideration the results of Liao et al. [13], Jin et al. [8] and our current study showing significantly decreased CSF Nogo-A and serum MAG concentrations in patients with brain tumour compared to non-tumoural individuals, it is tempting to hypothesize that the Nogo-A and MAG expression may influence primary brain tumour biology.

In our study, the Kaplan–Meier curve analysis showed that the probability of survival was significantly lower for subjects with lower CSF Nogo-A concentrations compared to those with higher CSF Nogo-A values. These results may indicate that decreased CSF Nogo-A concentration is an adverse prognostic factor for patient survival. Interestingly, Hao et al. [24] found that decreased expression of Nogo-A by SMMC 7721 hepatocellular carcinoma cell lines resulted in growth inhibition of these cells. A similar effect was not observed in the case of non-small cell lung carcinoma H1299 cell lines, colon carcinoma RKO cell lines, and ovarian cancer SKOV3 cell lines [24]. These results suggest that Nogo-A may exert diverse effects depending on the cancer cell type. This phenomenon could be explained by the differences in receptor complexes which may have a different array of constituents dependent upon the type of tumour cell. All NgR1 ligands: Nogo-A, MAG, and OMgp signal their effect through a receptor complex consisting of a minimum of Nogo-66 receptor. But most importantly, NgR1 is a GPI-linked protein and requires additional partners to the signal transduction [6].

In the next step, we conducted ROC analysis to investigate the accuracy of CSF Nogo-A and serum MAG concentration evaluation to differentiate patients with primary brain tumour (astrocytic brain tumours plus meningeal brain tumours) from non-tumoural individuals. The diagnostic utility analysis showed that the highest AUC (0.909), Youden Index (0.65), diagnostic specificity (100%), and positive predictive value (PPV) (100%) were found for CSF Nogo-A. Serum MAG displayed less worth in differentiating the total group of patients with primary brain tumour from non-tumoural individuals (AUC = 0.797, Youden Index = 0.57, diagnostic specificity = 80%). In other words, the evaluation of CSF Nogo-A concentrations was found to be the most suitable for primary brain tumour diagnosis, as the specificity of the test reached 100%. It is well-established that the higher the specificity, the smaller the number of false-positive results [25,26].

A deeper analysis of the NgR1 ligands found that CSF Nogo-A evaluation had lower diagnostic specificity (83%) compared to serum MAG (90%) when differentiating patients with astrocytic brain tumour from non-tumoural individuals. However, serum MAG had lower AUC (0.770), but higher PPV (93%) compared to CSF Nogo-A (0.871 and 86%, respectively). In the differential diagnosis of patients with meningeal brain tumour from non-tumoural individuals, CSF Nogo-A evaluation was the most appropriate test with AUC = 0.987 and a specificity of 100%. However, it should be noted that both PPV and negative predictive value (NPV) are likely to be interpreted depending on the prevalence of the disease [25,27].

The diagnostic utility analysis indicated the usefulness of CSF Nogo-A and serum MAG concentration evaluation to confirm primary brain tumour diagnosis. However, it is worth mentioning that in our case–control study the brain tumour group and the non-tumoural group were relatively small, thus estimated AUCs could be biased due to relatively few points on the curve. Other factors influencing the diagnostic accuracy analysis may be related to sampling bias, prevalence variability due to inappropriate study design, clinical patient variability, subgroup differences, or even reader expectations [25]. Undoubtedly, good management of any disease requires an accurate diagnosis of the patient, with minimal risk of misdiagnosis or missed diagnosis. Therefore, it is important to identify a diagnostic test that will be useful for specific conditions [25]. In this context, our results should be interpreted carefully and considered rather as a future direction helpful in the identification of specific primary brain tumour biomarkers.

Kaplan–Meyer curve analysis demonstrated that the probability of survival was significantly greater for females with higher CSF Nogo-A concentrations. This could be related to the fact that our previous study presented, utilizing multiple linear regression analysis, that the mean CSF Nogo-A concentration was 1.9 times higher for women in comparison to men [12]. Nevertheless, the relation between CSF Nogo-A concentration and patient survival requires further study.

It was also interesting for us to analyze how biochemical parameters routinely done for all patients with brain tumour may predict the probability of brain tumour diagnosis. Interestingly, multivariate logistic regression analysis showed that predictor variables influencing brain tumour diagnosis included: CSF Nogo-A, K+, urea, and creatinine concentration. There is no similar analysis in the available literature, which makes it impossible to discuss the obtained results. Undoubtedly, the fact that routinely evaluated biochemical parameters can predict the probability of brain tumour diagnosis is worth exploring in further studies.

One limitation of the study could be the sensitivity of the applied ELISA tests for Nogo-A, MAG, and OMgp concentration evaluation. The standard curve points of MAG and OMgp ELISA kits were expressed in ng/mL. On the contrary, for Nogo-A the standard curve points were expressed in pg/mL. These differences in the sensitivity of the ELISA kits may explain why some MAG and OMgp results were below the detection range. Another study limitation could be the specificity of the applied ELISA kits. Thus, we asked the ELISA kit suppliers for the anti-Nogo-A, anti-MAG, and anti-OMgp antibodies specificity. In the case of the Nogo-A test, the ELISA vendor used UniProtKB: Q9NQC (1-200aa) antibody, which is only human-specific. In the case of the MAG and OMgp tests, we have obtained information that the used kits did not reveal any cross-reactivity with myelin basic protein (MBP), myelin protein zero, and myelin protein two. The possible cross-reactivity of the OMgp ELISA kit with GPIbαβ was not tested.

It must be also acknowledged, that our “control group” did not include entirely healthy people, as they had diagnosed unruptured brain aneurysms. Nevertheless, despite this limitation, we still were able to demonstrate statistically significant differences. Moreover, there was a sex disparity in non-tumoural subjects, which was dictated by fact that female sex is a risk factor of brain aneurysms. Finally, to make a quantitative tumour marker clinically applicable for cancer diagnosis, it is necessary to establish reference values in the control group or evaluate a marker in a follow-up with a single patient [28]. None of these actions have been performed in the current study, which could also be considered a study limitation. But the current study was just a pilot study in nature, aiming to find potential circulating biomarkers for primary brain tumours.

## 5. Conclusions

We demonstrated significantly decreased CSF Nogo-A concentration in patients with primary brain tumour (astrocytic plus meningeal) compared to non-tumoural individuals. We also found that serum MAG concentration is significantly decreased in these patients compared to control subjects. The obtained results indicated CSF Nogo-A and serum MAG evaluation as potential circulating diagnostic markers of primary brain tumours. We believe that the study we conducted provides cognitive knowledge about quantitative myelin-associated protein evaluation in patients with primary brain tumour.

## Supplementary Material

Supplemental Material

## Data Availability

The datasets generated and/or analyzed during the current study are not publicly available, but are available from the corresponding author (OMK-L) on a request.

## Abbreviations

aa: amino acid
AUC: area under the ROC curve
BBB: blood–brain barrier
CNS: central nervous system
CR: Cys-rich motif
CSF: cerebrospinal fluid
Cys: cysteine
ELISA: enzyme-linked immunosorbent assay
GPI: glycosylphosphatidylinositol
GPIb: platelet glycoprotein Ib
IDH: isocitrate dehydrogenase
Leu: leucine
LRRs: Leucine-rich repeats
MAG: myelin-associated glycoprotein
MBP: myelin basic protein
NgR1: Nogo Receptor 1
Nogo-A: neurite growth isoform A of reticulon-4
OMgp: oligodendrocyte myelin glycoprotein
PirB: paired immunoglobulin receptor B protein
ROC: receiver operating characteristic
Ser: serine
Thr: threonine
WHO: World Health Organization.

## References

[1] Westphal M, Lamszus K. Circulating biomarkers for gliomas. Nat Rev Neurol. 2015;11(10):556–566. 26369507 10.1038/nrneurol.2015.171

[2] Albulescu R, Codrici E, Popescu ID, et al. Cytokine patterns in brain tumour progression. Mediators Inflamm. 2013;2013:979747–979748. 10.1155/2013/979748 PMC3707225 23864770

[3] Kros JM, Mustafa DM, Dekker LJM, et al. Circulating glioma biomarkers. Neuro Oncol. 2015;17(3):343–360. 25253418 10.1093/neuonc/nou207 PMC4483097

[4] Hochberg FH, Atai NA, Gonda D, et al. Glioma diagnostics and biomarkers: an ongoing challenge in the field of medicine and science. Expert Rev Mol Diagn. 2014;14(4):439–452. 24746164 10.1586/14737159.2014.905202 PMC5451266

[5] Qin X, Kang L, Liu Y, et al. Chinese medicine's intervention effect on Nogo-A/NgR. Evid Based Complement Alternat Med. 2012; 2012:528482–528484. 22216056 10.1155/2012/528482 PMC3247900

[6] McKerracher L, Rosen KM. MAG, myelin and overcoming growth inhibition in the CNS. Front Mol Neurosci. 2015;8:51–56. 26441514 10.3389/fnmol.2015.00051 PMC4561339

[7] Fernández-Suárez D, Krapacher FA, Andersson A, et al. MAG induces apoptosis in cerebellar granule neurons through p75NTR demarcating granule layer/white matter boundary. Cell Death Dis. 2019;10(10):732. 31570696 10.1038/s41419-019-1970-x PMC6768859

[8] Jin S-G, Ryu H-H, Li S-Y, et al. Nogo-A inhibits the migration and invasion of human malignant glioma U87MG cells. Oncol Rep. 2016;35(6):3395–3402. 27109183 10.3892/or.2016.4737

[9] Marucci G, Di Oto E, Farnedi A, et al. Nogo-A: a useful marker for the diagnosis of oligodendroglioma and for identifying 1p19q codeletion. Hum Pathol. 2012;43(3):374–380. 21835431 10.1016/j.humpath.2011.05.007

[10] Jung T-Y, Jung S, Lee K-H, et al. Nogo-A expression in oligodendroglial tumors. Neuropathology. 2011;31(1):11–19. 20487307 10.1111/j.1440-1789.2010.01118.x

[11] Kuhlmann T, Gutenberg A, Schulten H-J, et al. Nogo-A expression in glial CNS tumors: a tool to differentiate between oligodendrogliomas and other gliomas? Am J Surg Pathol. 2008;32(10):1444–1453. 18685489 10.1097/PAS.0b013e31817ce978

[12] Koper OM, Kamińska J, Milewska A, et al. The isoform A of reticulon-4 (Nogo-A) in cerebrospinal fluid of primary brain tumor patients: influencing factors. Oncotarget. 2018;9:25048–25056. 29861852 10.18632/oncotarget.25278 PMC5982740

[13] Liao H, Duka T, Teng FYH, et al. Nogo-66 and myelin-associated glycoprotein (MAG) inhibit the adhesion and migration of nogo-66 receptor expressing human glioma cells. J Neurochem. 2004;90(5):1156–1162. 15312170 10.1111/j.1471-4159.2004.02573.x

[14] Cebral J, Ollikainen E, Chung BJ, et al. Flow conditions in the intracranial aneurysm lumen are associated with inflammation and degenerative changes of the aneurysm wall. AJNR Am J Neuroradiol. 2017;38(1):119–126. 27686488 10.3174/ajnr.A4951 PMC5233582

[15] Kamińska J, Lyson T, Chrzanowski R, et al. Ratio of IL-8 in CSF versus serum is elevated in patients with unruptured brain aneurysm. J Clin Med. 2020;9(6):1761. 32517149 10.3390/jcm9061761 PMC7356854

[16] Koper OM, Kamińska J, Sawicki K, et al. Cerebrospinal fluid and serum IL-8, CCL2, and ICAM-1 concentrations in astrocytic brain tumor patients. Ir J Med Sci. 2018;187(3):767–775. 29086194 10.1007/s11845-017-1695-8

[17] Shan G. Improved confidence intervals for the youden index. PLoS One. 2015;10(7):e0127272. 26132806 10.1371/journal.pone.0127272 PMC4488538

[18] Hajian-Tilaki K. Receiver operating characteristic (ROC) curve analysis for medical diagnostic test evaluation. Casp J Intern Med. 2013;4:627–635. http://www.ncbi.nlm.nih.gov/pubmed/24009950. PMC3755824 24009950

[19] Lai C-Y, Tian L, Schisterman EF. Exact confidence interval estimation for the youden index and its corresponding optimal cut-point. Comput Stat Data Anal. 2012;56(5):1103–1114. 27099407 10.1016/j.csda.2010.11.023 PMC4834986

[20] Villa C, Miquel C, Mosses D, et al. The 2016 world health organization classification of tumours of the Central nervous system. Presse Med. 2018;47(11–12 Part 2):e187–e200. 30449638 10.1016/j.lpm.2018.04.015

[21] Horbinski C. What do we know about IDH1/2 mutations so far, and how do we use it? Acta Neuropathol. 2013;125(5):621–636. 23512379 10.1007/s00401-013-1106-9 PMC3633675

[22] Mikol DD, Alexakos MJ, Bayley CA, et al. Structure and chromosomal localization of the gene for the oligodendrocyte-myelin glycoprotein. J Cell Biol. 1990;111(6 Part 1):2673–2679. 2277079 10.1083/jcb.111.6.2673 PMC2116377

[23] Buss A, Sellhaus B, Wolmsley A, et al. Expression pattern of NOGO-A protein in the human nervous system. Acta Neuropathol. 2005;110(2):113–119. 15616791 10.1007/s00401-004-0942-z

[24] Hao C-Q, Zhou Y, Wang J-P, et al. Role of Nogo-A in the regulation of hepatocellular carcinoma SMMC7721 cell apoptosis . Mol Med Rep. 2014;9(5):1743–1748. 24626842 10.3892/mmr.2014.2050

[25] Bolboacă SD. Medical diagnostic tests: a review of test anatomy, phases, and statistical treatment of data. Comput Math Methods Med. 2019;2019:1891569–1891522. 31275427 10.1155/2019/1891569 PMC6558629

[26] Parikh R, Mathai A, Parikh S, et al. Understanding and using sensitivity, specificity and predictive values. Indian J Ophthalmol. 2008;56(1):45–50. 18158403 10.4103/0301-4738.37595 PMC2636062

[27] Trevethan R. Sensitivity, specificity, and predictive values: Foundations, pliabilities, and pitfalls in research and practice. Front Public Health. 2017; 5:307. 29209603 10.3389/fpubh.2017.00307 PMC5701930

[28] Wagner PL, Austin F, Sathaiah M, et al. Significance of serum tumor marker levels in peritoneal carcinomatosis of appendiceal origin. Ann Surg Oncol. 2013;20(2):506–514. 22941175 10.1245/s10434-012-2627-5 PMC3812816

