# Supplementary material for: Myelin-associated proteins are potential diagnostic markers in patients with primary brain tumour
Source: Ann Med. 2021 Oct 3;53(1):1710–21. doi: 10.1080/07853890.2021.1983205 (PMC8491661; doi:10.1080/07853890.2021.1983205)
Supplement: Supplemental Material [file IANN_A_1983205_SM1846.docx]

**Table S1.** Characteristics of individual brain tumor patients.

| **No.** | **Histopathological**  **examination** | | **Sex** | **Age** | **WHO** | **IDH1** |
| --- | --- | --- | --- | --- | --- | --- |
|  | | **Astrocytic brain tumor patients** | | | | |
| **1** | Diffuse astrocytoma | | M | 39 | 2 | (-) |
| **2** | Glioblastoma | | M | 45 | 4 | (-) |
| **3** | Glioblastoma | | F | 67 | 4 | (-) |
| **4** | Anaplastic astrocytoma | | M | 60 | 4 | (-) |
| **5** | Diffuse astrocytoma | | F | 70 | 2 | (-) |
| **6** | Glioblastoma | | M | 41 | 4 | (+) |
| **7** | Glioblastoma | | F | 73 | 4 | (+) |
| **8** | Glioblastoma | | F | 72 | 4 | (+) |
| **9** | Glioblastoma | | M | 58 | 4 | (+) |
| **10** | Glioblastoma | | M | 55 | 4 | (+) |
| **11** | Anaplastic glioma | | M | 57 | 3 | (-) |
| **12** | Glioblastoma | | F | 44 | 4 | (-) |
| **13** | Glioblastoma | | M | 57 | 4 | (+) |
| **14** | Pilocytic astrocytoma | | M | 42 | 1 | (-) |
| **15** | Glioblastoma | | F | 65 | 4 | (-) |
| **16** | Glioblastoma | | M | 59 | 4 | (-) |
| **17** | Glioblastoma | | F | 51 | 4 | (+) |
| **18** | Anaplastic astrocytoma | | F | 40 | 3 | (-) |
| **19** | Glioblastoma | | M | 72 | 4 | (-) |
| **20** | Glioblastoma | | F | 62 | 4 | (-) |
| **21** | Glioblastoma | | M | 44 | 4 | (+) |
| **22** | Glioblastoma | | M | 43 | 4 | (+) |
| **23** | Glioblastoma | | F | 59 | 4 | (-) |
| **24** | Glioblastoma | | M | 69 | 4 | (+) |
| **25** | Glioblastoma | | M | 67 | 4 | (-) |
| **26** | Glioblastoma | | M | 73 | 4 | (-) |
| **27** | Glioblastoma | | M | 63 | 4 | (+) |
| **28** | Glioblastoma | | M | 66 | 4 | (+) |
| **29** | Glioblastoma | | M | 48 | 4 | (+) |
| **30** | Glioblastoma | | M | 69 | 4 | (-) |
| **31** | Glioblastoma | | F | 59 | 4 | (-) |
| **32** | Glioblastoma | | M | 59 | 4 | (-) |
| **33** | Glioblastoma | | M | 58 | 2 | (+) |
|  | | **Meningeal brain tumor patients** | | | | |
| **1** | Transitional meningioma with psammoma bodies | | F | 68 | 1 | Not applicable |
| **2** | Psammomatous meningioma | | F | 45 | 1 |  |
| **3** | Transitional meningioma | | F | 46 | 1 |  |
| **4** | Transitional meningioma | | M | 83 | 1 |  |
| **5** | Meningothelial meningioma | | F | 47 | 1 |  |
| **6** | Fibroblastic meningioma | | F | 62 | 1 |  |
| **7** | Psammomatous meningioma | | F | 72 | 1 |  |
| **8** | Anaplastic meningioma | | M | 58 | 3 |  |
| **9** | Meningothelial meningioma | | F | 36 | 1 |  |
| **10** | Meningothelial meningioma | | F | 64 | 1 |  |
| **11** | Transitional meningioma | | F | 60 | 1 |  |
| **12** | Meningothelial meningioma | | F | 70 | 1 |  |
| **13** | Transitional meningioma | | F | 75 | 1 |  |
| **14** | Atypical meningioma | | M | 43 | 2 |  |
| **15** | Meningothelial meningioma | | F | 41 | 1 |  |
| **16** | Meningothelial meningioma | | F | 76 | 1 |  |

**Legend to the Table S1:** WHO – World Health Organization, IDH1 – isocitrate dehydrogenase 1, M – male, F – female.
